# Assessing the Impact of Lymphedema Therapy Referral on Breast Cancer Survivors’ Lymphedema Knowledge: A Cross-Sectional Survey

**DOI:** 10.1186/s12905-025-03654-x

**Published:** 2025-03-15

**Authors:** Madelyn Klugman, Yashasvini Sampathkumar, Sujata Patil, Kathryn R. Tringale, Giacomo Montagna, Jackie Finik, Ting-Ting Kuo, Carolyn Eberle, Alexandr Pinkhasov, Theresa Gillis, Francesca Gany, Victoria Blinder

**Affiliations:** 1 The Sidney Kimmel Comprehensive Cancer Center at Johns Hopkins, Baltimore, MD USA; 2 Department of Epidemiology and Biostatistics, Memorial Sloan Kettering Cancer Center, New York, NY USA; 3 Quantitative Health Sciences Department, Cleveland Clinic, Cleveland, OH USA; 4 Department of Radiation Medicine and Applied Sciences, University of California, San Diego School of Medicine, San Diego, CA USA; 5 Breast Service, Department of Surgery, Memorial Sloan Kettering Cancer Center, New York, NY USA; 6 Immigrant Health and Cancer Disparities Service, Department of Psychiatry and Behavioral Sciences, Memorial Sloan Kettering Cancer Center, New York, NY USA; 7 Rehabilitation Medicine Service, Department of Neurology, Memorial Sloan Kettering Cancer Center, New York, NY USA; 8 Department of Epidemiology, UNC Gillings School of Public Health, Chapel Hill, NC USA; 9 Department of Urology, SUNY Upstate University Hospital, Syracuse, NY USA; 10 Breast Medicine Service, Department of Medicine, Memorial Sloan Kettering Cancer Center, New York, NY USA

**Keywords:** Disability, Physical therapy, Survivorship, Symptom management

## Abstract

**Background:**

Lymphedema is a common problem that adversely impacts quality of life in breast cancer survivors. Although lymphedema risk is modifiable through behavior change, there is no standardized approach to educate survivors about risk-lowering strategies. Furthermore, misconceptions about lymphedema risk factors and risk-lowering strategies are common. The aim of this study was to evaluate the effect of lymphedema therapy referral on knowledge about lymphedema risk.

**Methods:**

This was a cross-sectional single institution study in which breast cancer survivors at a National Cancer Institute-designated cancer center completed an anonymous questionnaire between 2014 and 2015. Eligibility criteria were age ≥ 18, female sex, English-speaking, > 6 months post definitive breast cancer surgery, no cancer recurrence, and no prior or subsequent second cancer. The questionnaire included sociodemographic variables, clinical factors including prior lymphedema therapy referral, and 10 true/false questions assessing knowledge about lymphedema risk. Multivariable logistic regression analyses assessed the relationship between prior lymphedema therapy referral and correctly answering questions about lymphedema risk.

**Results:**

Of 209 participants, 53 (25%) had been referred to lymphedema therapy. Those who had undergone sentinel lymph node biopsy were less frequently referred to lymphedema therapy [15 (14%)] than those who had undergone axillary lymph node dissection [38 (39%)]. Five of the true/false questions had a correct response rate of < 80%. After controlling for age, race/ethnicity, education, type of axillary surgery, and receipt of radiation therapy, referral for lymphedema therapy was associated with correctly answering two questions about lymphedema: weight gain increases lymphedema risk [odds ratio, 95% confidence interval: 3.63 (1.66–7.96)] and patients are recommended to exercise their arm on an airplane [2.65 (1.15–6.13)].

**Conclusions:**

Misconceptions about lymphedema prevention and management are common among breast cancer survivors. Lymphedema therapy referral is a potential opportunity to debunk misunderstandings and educate at-risk patients regarding lymphedema.

**Supplementary Information:**

The online version contains supplementary material available at 10.1186/s12905-025-03654-x.

## Background

Lymphedema, which can cause swelling, discomfort, and impaired arm function, is an important source of morbidity among breast cancer survivors. The estimated risk of developing lymphedema after treatment for breast cancer is approximately 20% [1–3], with the main risk factor being axillary lymph node dissection [4, 5].

Given this pervasive morbidity, the 2022 National Comprehensive Cancer Network guidelines note that early detection and diagnosis of lymphedema is vital, and breast cancer care providers should “educate, monitor, and refer for lymphedema management” [6]. Furthermore, the National Lymphedema Network has issued guidelines to prevent the development and exacerbation of lymphedema [7], which relate to both exercise and modifications to daily activities, including that “exercise is recommended for those with and at risk for lymphedema” and “exercise should be started gradually, increased cautiously, and stopped for pain, increased swelling, or discomfort.” These recommendations are based on the literature demonstrating that exercise does not increase the risk of lymphedema [8, 9] and may instead, as part of a multimodal approach, reduce the risk of chronic lymphedema or exacerbation of established lymphedema [10]. Nevertheless, misconceptions regarding lymphedema prevention and management are pervasive, particularly regarding using the arm for exercise and for common activities (e.g., carrying groceries) [11, 12]. Of note, the guidelines regarding activities of daily living mainly concern limiting stasis (e.g., exercising the arm on an airplane) and infectious exposures (e.g., covering the affected arm while gardening to avoid punctures), not weight-bearing precautions [13]. Therefore, breast cancer survivors who limit use of their affected arm following surgery based on these misconceptions may be imposing an unnecessary burden on themselves (e.g., if these limitations impact their ability to work) and may actually be increasing their risk of developing or exacerbating lymphedema [2].

The purpose of this study was to describe the characteristics of breast cancer survivors who were referred for lymphedema therapy, and to understand if patients who have previously been referred for lymphedema therapy have fewer misconceptions about lymphedema than those who have never been referred.

## Methods

### Data source and study population

In this cross-sectional study, breast cancer survivors were approached during follow-up visits in the waiting room at Memorial Sloan Kettering Cancer Center between 2014 and 2015 and asked to complete an anonymous paper survey. The eligibility criteria included age ≥ 18 years, female sex, English-speaking, completed definitive surgery for breast cancer (including axillary lymph node dissection or sentinel lymph node biopsy) at least 6 months before time of survey, had no cancer recurrence, and no prior or subsequent second cancer. The protocol was approved as Institutional Review Board-exempt and qualified for a waiver of written informed consent under federal regulations from the United States Department of Health and Human Services, Office for Human Research Protections.

### Measures and outcomes

Information about type of axillary surgery [sentinel lymph node biopsy (SLNB) or axillary lymph node dissection (ALND)] was abstracted from the medical record at the time of recruitment, before the participant completed the survey, and documented by study staff on the participant’s paper survey form. Medical records were not otherwise accessed. All other variables were self-reported, including age, education (stratified by less than a bachelor’s degree and at least a bachelor’s degree), race/ethnicity, and breast cancer treatment (other than type of axillary surgery). Participants were also asked to report if they had been referred to lymphedema therapy, if they had been told that they had a diagnosis of lymphedema by a member of their clinical team, and what their main sources had been for information about arm and hand edema (e.g., doctors, nurses, lymphedema therapists, pamphlets, internet, friends, support group). For the latter question, participants were allowed to select multiple options.

Finally, a lymphedema knowledge survey (Appendix 1) was administered. The survey was developed by lymphedema therapists and a medical oncologist based on their expertise, a review of the literature [16], and the National Lymphedema Network (NLN) guidelines [7]; it assesses lymphedema knowledge via ten true/false questions concerning prevention of lymphedema development or progression.

### Statistical analysis

Descriptive statistics were used to compare demographic characteristics, clinical factors, and lymphedema knowledge (i.e., proportion of questions answered correctly) between those referred for lymphedema therapy and those who were not referred. The chi-squared test was used for categorical comparisons and the Wilcoxon rank-sum test was used for continuous variables (time from diagnosis to time of survey).

Univariate logistic regression was first used to assess factors related to answering the lymphedema knowledge questions with < 80% correct response rates. Then, a multivariable analysis assessed the association between lymphedema therapy referral and lymphedema knowledge questions. Models were adjusted for sociodemographic characteristics and the breast cancer treatments most associated with lymphedema risk [ALND and radiation therapy (RT)] [1]. Goodness-of-fit testing was performed, with each model having a p-value for the Hosmer-Lemshow chi-squared test > 0.05, suggesting a good fit. In each of the models, fewer than 25% of the expected frequencies were less than 5, suggesting the chi-squared model was appropriate to use.

For all statistical analyses, unless otherwise mentioned, values were considered statistically significant if *p* < 0.05. The statistical software Stata, version 15.1, was used for all analyses.

## Results

### Patient characteristics, overall and by lymphedema referral status

Of 209 participants who completed the survey, 110 participants (53%) were at least 55 years old. Regarding race and ethnicity, 17 (8%) identified as Asian, 27 (13%) as Black, 21 (10%) as Latina, 138 (67%) as non-Latina white, and 4 (2%) as mixed race or other.

Overall, 53 participants (25%) were referred for lymphedema therapy. Most participants who were referred for lymphedema therapy reported that they received most of their knowledge about lymphedema from a lymphedema therapist. Bivariate comparisons between those referred for lymphedema therapy and those not referred revealed that those who were told by their clinical team that they had lymphedema and those who received more intensive treatments (i.e., ALND, RT, and/or chemotherapy) were more likely to be referred for lymphedema therapy (Table 1). However, length of time since diagnosis did not differ by referral status [3.3 years (IQR 1.8–5.2) in those not referred versus 2.7 years (2.1–4.7) in those referred; *p* = 0.75].

**Table 1:** Descriptive characteristics of 209 breast cancer survivors surveyed about lymphedema

	Not referred to lymphedema therapy, *n* = 156 No. (%)	Referred to lymphedema therapy, *n* = 53 No. (%)	*p*-value
**Age**			0.02
18–44	32 (21)	17 (32)	
45–54	41 (26)	9 (17)	
55–64	38 (24)	20 (38)	
65+	45 (29)	7 (13)	
**Education**			0.67
< Bachelor’s	55 (35)	17 (32)	
Bachelors+	101 (65)	36 (68)	
**Race/Ethnicity**			0.46
Asian	15 (10)	2 (4)	
Black	19 (12)	8 (15)	
Hispanic/Latina	16 (10)	5 (9)	
White	100 (65)	38 (72)	
Mixed or other	4 (3)	0 (0)	
**Patient-reported lymphedema diagnosis**			< 0.01
No	142 (91)	21 (40)	
Yes	13 (8)	32 (60)	
**Nodal procedure**			< 0.01
Sentinel lymph node biopsy	96 (62)	15 (28)	
Axillary lymph node dissection	60 (38)	38 (72)	
**Radiation therapy**			< 0.01
No	58 (37)	7 (13)	
Yes	98 (63)	46 (87)	
**Chemotherapy**			< 0.01
No	57 (37)	7 (13)	
Yes	99 (63)	46 (87)	
**Surgery**			0.41
Lumpectomy	73 (48)	22 (42)	
Mastectomy	79 (52)	31 (58)	
Time from survey to diagnosis [years, median (IQR)]	3.3 (1.8–5.2)	2.7 (2.1–4.7)	0.75
Lymphedema therapist is info source*	5 (3)	37 (70)	< 0.01
Doctor is info source*	77 (49)	20 (38)	0.14
Nurse is info source*	36 (23)	10 (19)	0.52
**Friends**,** internet**, or support group are info source*	38 (24)	19 (35)	0.11

*Participants allowed to select multiple options

### Lymphedema knowledge by lymphedema referral status

The percentage of participants who correctly answered each lymphedema knowledge question ranged from 48 to 94% (Table 2). Five of the ten lymphedema knowledge questions had a correct response rate of < 80%. Participants who were referred for lymphedema therapy were less likely to correctly answer the questions about using the arm on the side of breast surgery for strenuous activity at work (33% versus 52%, *p* = 0.02) or for carrying greater than ten pounds (32% versus 54%, *p* < 0.01). On the other hand, those referred for lymphedema therapy were more likely to correctly identify weight gain as a factor associated with increased lymphedema risk (73% versus 49%, *p* < 0.01) and exercising the arm while on an airplane as a risk-mitigating factor (80% versus 58%, *p* < 0.01). Five questions were answered correctly by > 80% of the sample; these questions involved resting the affected arm as much as possible, exercising and stretching the arm regularly, waiting to call the doctor for arm redness/swelling, and avoiding using the arm for chores or light office work. For these five questions, there were no differences in lymphedema knowledge by lymphedema referral status.

**Table 2:** Proportion of breast cancer survivors who correctly answered questions about common lymphedema misconceptions, by referral to lymphedema therapy

Question (correct answer)	Total patients (*N* = 209) with correct answer, No. (%)	Patients NOT referred to lymphedema therapy (*n* = 156) with correct answer, No. (%)	Patients referred to lymphedema therapy (*n* = 53) with correct answer, No. (%)	*p*-value
Avoid using arm on affected side for strenuous activity at work (False)	97 (48)	80 (52)	17 (33)	0.02
Avoid using arm on affected side to carry > 10lbs (False)	100 (49)	84 (54)	16 (32)	< 0.01
Weight gain can increase risk of lymphedema (True)	112 (55)	75 (49)	37 (73)	< 0.01
Exercise arm on airplane (True)	128 (63)	88 (58)	40 (80)	< 0.01
Avoid using arm on affected side to lift weights (False)	151 (75)	112 (74)	39 (76)	0.74
Rest arm as much as possible (False)	169 (82)	128 (83)	41 (80)	0.72
Exercise and stretch arm regularly (True)	179 (86)	131 (84)	48 (94)	0.07
Wait a day to call doctor if arm becomes red/warm (False)	189 (92)	142 (92)	47 (92)	0.90
Avoid using arm for house chores (False)	191 (92)	142 (91)	49 (96)	0.24
Avoid using arm for light office work (False)	196 (95)	148 (95)	48 (94)	0.84

### Factors related to correct lymphedema knowledge

For the five questions that were answered correctly by < 80% of participants, univariate logistic regressions were used to identify characteristics associated with knowing the correct answer (Table 3). Age, race/ethnicity, and receipt of RT were not associated with correctly answering the lymphedema knowledge questions.

**Table 3:** Univariate analysis of factors related to correctly answering questions about lymphedema risk (Sample size range for each item is 199–204, with < 5% missing data.)

	Avoid using arm for strenuous activity		Avoid using arm on affected side to carry > 10lbs		Weight gain can increase risk of lymphedema		Exercise arm on airplane		Avoid using arm on affected side to lift weights	
	OR	95% CI	OR	95% CI	OR	95% CI	OR	95% CI	OR	95% CI
**Lymphedema therapy referral**	**0.46**	**0.24–0.89**	**0.4**	**0.21–0.79**	**2.71**	**1.36–5.42**	**2.91**	**1.35–6.25**	1.13	0.54–2.38
**Age**										
18–44	1	ref	1	ref	1	ref	1	ref	1	ref
45–54	1.50	0.68–3.33	0.96	0.43–2.11	1.97	0.87–4.45	0.65	0.29–1.47	0.98	0.35–2.71
55–64	0.59	0.27–1.31	0.61	0.28–1.32	2.13	0.97–4.68	1.34	0.58–3.06	0.66	0.26–1.69
65+	1.09	0.50–2.38	0.62	0.28–1.36	1.05	0.47–2.32	0.92	0.41–2.10	0.37	0.15–0.92
**Education**										
< Bachelor’s	1	ref	1	ref	1	ref	1	ref	1	ref
Bachelors+	**2.26**	**1.25–4.09**	**2.62**	**1.44–4.76**	1.21	0.68–2.16	**1.98**	**1.09–3.60**	**2.26**	**1.18–4.34**
**Race/Ethnicity**										
Non-Latina white	1	ref	1	ref	1	ref	1	ref	1	ref
Black	0.28	0.11–0.73	0.37	0.15–0.91	0.81	0.35–1.85	0.87	0.37–2.05	0.47	0.19–1.34
Latina	0.65	0.25–1.68	0.59	0.23–1.54	0.50	0.19–1.30	0.42	0.16–1.08	1.24	0.39–3.99
Asian	2.33	0.78–6.98	1	0.36–2.75	1.07	0.38–2.99	1.66	0.51–5.39	1.45	0.39–5.37
Mixed or other	2.91	0.29–28.71	0.89	0.12–6.49	2.25	0.23–22.20	0.17	0.02–1.69	0.31	0.04–2.29
**Nodal Procedure**										
Sentinel lymph node biopsy	1	ref	1	ref	1	ref	1	ref	1	ref
Axillary lymph node dissection	**0.54**	**0.31–0.95**	0.59	0.34–1.03	1.06	0.61–1.84	1.74	0.97–3.13	1.66	0.87–3.19
**Radiation therapy**	1.04	0.58–1.88	0.92	0.51–1.66	0.88	0.48–1.61	1.28	0.70–2.36	1.49	0.76–2.91
**Surgery**										
Lumpectomy	1	ref	1	ref	1	ref	1	ref	1	ref
Mastectomy	**0.55**	**0.31–0.96**	0.65	0.37–1.13	1.12	0.64–1.97	1.19	0.67–2.12	0.99	0.52–1.88
**Chemotherapy**	0.55	0.30-1.00	0.58	0.32–1.06	1.35	0.74–2.46	1.2	0.65–2.23	1.08	0.54–2.14
**Info from lymphedema therapist?**	0.50	0.24–1.02	0.65	0.32–1.31	**2.30**	**1.10–4.82**	**3.32**	**1.39–7.96**	1.25	0.55–2.85
Info from other trusted clinical sources*	1.31	0.75–2.29	1.19	0.67–2.08	**0.54**	**0.31–0.96**	**0.34**	**0.18–0.63**	0.95	0.50–1.81

*Doctor, nurse, pamphlet, physical therapy, occupational therapy

Statistically significant results in bold

Odds Ratio (OR) and 95% confidence interval (95% CI) of answering question correctly

Higher educational attainment was associated with knowing the correct answer for four of these questions (all except “weight gain can increase risk of lymphedema”). Participants who had undergone ALND were less likely to correctly answer the question about using the arm for strenuous activity than those who had undergone SLNB (OR = 0.54, 95% CI: 0.31–0.95). Similarly, participants who had undergone mastectomy were less likely to correctly answer the question about using the arm for strenuous activity than those who had undergone lumpectomy (OR = 0.55, 95% CI: 0.31–0.96).

Participants who reported that their main source of lymphedema knowledge was a lymphedema therapist more likely to answer correctly regarding the questions about weight gain increasing lymphedema risk and exercising the arm on an airplane.

### Relationship between lymphedema therapy referral and lymphedema knowledge

**Fig. 1:**
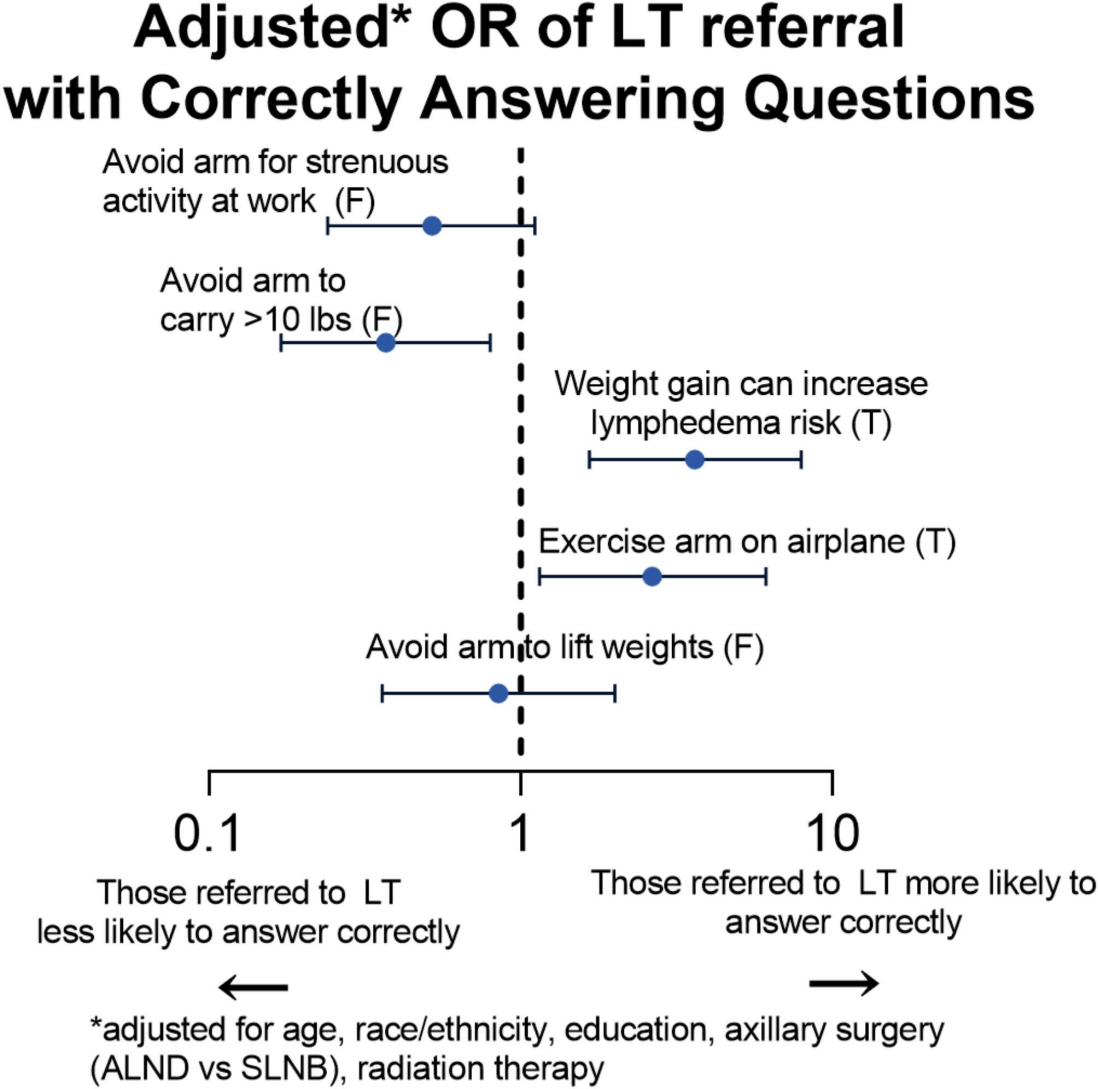
Odds of correctly answering questions about lymphedema misconceptions by lymphedema therapy referral status, adjusted for age, race/ethnicity, education, axillary surgery (ALND vs. SLNB), and radiation therapy. Abbreviations - F: False, T: True

In multivariable logistic regression, after adjusting for age, race/ethnicity, education, type of axillary surgery (ALND vs. SLNB), and receipt of RT, lymphedema therapy referral continued to be significantly associated with answering the questions about weight gain and exercising the arm on an airplane correctly [weight gain: OR = 3.63, 95% CI (1.66–7.96); airplane: OR = 2.65, 95% CI (1.15–6.13) Fig. 1]. Lymphedema therapy referral was also associated with incorrectly answering the question about carrying greater than ten pounds, but no longer associated with answering the question about strenuous activity incorrectly. Higher education (at least a bachelor’s degree) was associated with correctly answering questions about strenuous activity and carrying greater than ten pounds [strenuous: OR (95% CI) = 2.23 (1.10–4.50); ten pounds: OR (95% CI) = 2.44 (1.22–4.88)]. Other sociodemographic and treatment factors were not associated with answering questions correctly.

## Discussion

In this study, we found several lymphedema misconceptions that are pervasive among breast cancer survivors, similar to prior reports [14, 15]. Survivors commonly held misunderstandings about the role of exercising the affected arm and failed to recognize weight gain as a risk factor for lymphedema. Breast cancer survivors who were referred for lymphedema therapy demonstrated a better understanding of several (though not all) lymphedema risk concepts compared to those not referred, even in models adjusted for clinical and sociodemographic factors. Lymphedema therapy referral is an actionable step that providers can take to improve patients’ understanding of lymphedema risk, with the goal of preventing lymphedema development and progression.

We found that receiving RT and/or chemotherapy was not associated with answering the questions correctly. This suggests frequent clinician visits during treatment do not lead to improved lymphedema knowledge. Similarly, patients who received information about lymphedema from sources other than a lymphedema therapist were not found to have improved lymphedema knowledge. This highlights the importance of referral to a lymphedema therapist as a distinct component of clinical care. If lymphedema therapy is not readily accessible, focusing on increasing lymphedema education by other qualified providers could be considered. Finally, higher educational attainment was associated with answering most of the lymphedema knowledge questions correctly. Additional resources are needed to improve knowledge about lymphedema risk and prevention among patients with lower formal educational attainment.

We found lymphedema therapy referral to be associated with incorrectly answering a question about using the affected arm to carry greater than ten pounds, which may be due to the question’s wording. Lymphedema therapists generally prescribe progressive exercise and activity levels, and participants (and perhaps their lymphedema therapist) may have thought ten pounds was too heavy for an initial weight. The recommendation to start at lower weight and prescribe progressive exercise is based on randomized clinical trials of weightlifting/progressive resistive exercise for lymphedema prevention and management; these recommendations generally start with minimal resistance and gradually progress with no limits on maximal weight [2, 16, 17]. Furthermore, if a lymphedema exacerbation occurs, the upper extremity exercises restart at minimal weight [2]. Of note, some studies have shown no lymphedema exacerbations and higher quality of life in women who are at risk for or who have a diagnosis of lymphedema and are undergoing progressive high-load exercise programs [18–20]. Our study indicates a need for all clinicians, including lymphedema therapists, to clearly explain the recommendations regarding exercise and weight-bearing to prevent the unnecessary imposition of restrictions that could adversely impact patients.

While prior studies have assessed lymphedema knowledge among cancer survivors [21, 22], our study is novel in its focus on the prevalence of lymphedema therapy referral as a key factor in determining knowledge of lymphedema risk. A systematic review assessed the association between lymphedema education and breast cancer outcomes related to function, quality of life, and lymphedema [14]. Only one trial assessed knowledge levels after implementation of an educational intervention. However, this study assessed general knowledge of breast cancer care rather than focusing on lymphedema [23]. A recent cross-sectional survey [22] also found that many breast cancer survivors have misconceptions regarding risk of developing lymphedema, but the study did not compare knowledge within different groups, such as those referred for lymphedema therapy, a key strength of our study.

Additional strengths of this study include its use of a straightforward survey easily completed in waiting rooms. Our results have clinical implications, highlighting the benefits of lymphedema therapy referrals in enhancing knowledge among breast cancer survivors. Furthermore, we had a diverse study sample, which allowed us to account for important sociodemographic and clinical factors in our multivariable analysis. In particular, the inclusion of all participants who had breast cancer surgery, not just those at high risk of lymphedema, allowed for comparisons of knowledge based on clinical risk factors and adds to the generalizability of our findings.

A limitation of this study is that the 10-item lymphedema knowledge questionnaire includes an item about exercising the arm during air travel as a risk-reduction behavior. We recognize that recent studies have demonstrated that air travel is not a significant risk factor for breast cancer-related lymphedema onset [24] or exacerbation [25]. However, this recommendation is still listed in the NLN guidelines [7] and was included in the questionnaire. Therefore, we have included this item in our analysis.

This analysis also lacks information on why participants were referred to lymphedema therapy. Reasons for referral could include clinical evidence of lymphedema, or high clinical risk profile due to high BMI or high number of axillary lymph nodes removed. Changes in standard surgical practices in the last several years have decreased the use of ALND in clinical practice [26]. We do not know if participants carried a formal diagnosis of lymphedema (documented in the medical record) as the data were collected through an anonymous survey. Our analysis showed that 45 participants (22%) recalled having been told they had lymphedema by their clinical team, indicating that our sample includes some participants at risk of developing lymphedema and others with preexisting lymphedema at risk of exacerbation. Another limitation is the lack of information regarding whether participants who were referred for lymphedema therapy had health insurance that would cover the therapy. Notably, 70% of participants who were referred for lymphedema therapy cited lymphedema therapists as a main source of information, suggesting that most participants who were referred were able to meet with a lymphedema therapist. Finally, as this study was conducted through an anonymous waiting room survey, we are unable to ascertain the total number of people who were approached and declined participation.

Future research is needed to determine if improved lymphedema knowledge is associated with improved lymphedema outcomes in breast cancer survivors. In addition, research is needed regarding improving lymphedema knowledge among high-risk patients in resource-limited settings. Lymphedema therapy is not always readily available, and additional reliable modes of patient education should be investigated. In those cases, providers who are not lymphedema therapists might benefit from training in evidence-based guidelines on risk reduction of lymphedema [24, 25]. A proactive approach to minimizing lymphedema risk through appropriate education during the periods surrounding surgery and radiation could help patients better understand and mitigate their risk without unnecessarily restricting their activities.

## Conclusions

Our findings indicate that misconceptions about lymphedema risk are widespread among patients who have undergone breast cancer surgery. Some of these misconceptions may be mitigated by referral to a lymphedema therapist. Additional efforts are needed to improve educational outreach and the effective transmission of correct information about lymphedema risk. These efforts should focus on accurately describing steps patients can take to mitigate their risk without incorrectly imposing restrictions that are, at best, unnecessary and, at worst, harmful.

## Electronic supplementary material

Below is the link to the electronic supplementary material.

Appendix 1

## Data Availability

The data described in the current study are available from the corresponding author on reasonable request.

## Declarations

### Abstract word count

293.

### Manuscript word count

2631.

## Abbreviations

SLNB: sentinel lymph node biopsy
ALND: axillary lymph node dissection
NLN: National Lymphedema Network
RT: radiation therapy
IQR: interquartile range
