# Supplementary material for: Assessing the Impact of Lymphedema Therapy Referral on Breast Cancer Survivors’ Lymphedema Knowledge: A Cross-Sectional Survey
Source: BMC Womens Health. 2025 Mar 15;25:123. doi: 10.1186/s12905-025-03654-x (PMC11909997; doi:10.1186/s12905-025-03654-x)
Supplement: Supplementary file 1 — Appendix 1 [file 12905_2025_3654_MOESM1_ESM.docx]

**Appendix 1.** Lymphedema Knowledge Survey

| *In order to prevent* *lymphedema* ***OR*** *keep it from getting worse . . .* | | |
| --- | --- | --- |
| **TRUE** | **FALSE** |  |
|  |  | I should not use the arm on the side of my breast surgery to carry more than 10 pounds. |
|  |  | I should exercise my arm while I am flying in an airplane. |
|  |  | I should rest my arm on the side of my breast surgery as much as possible. |
|  |  | I should not use the arm on the side of my breast surgery to lift weights at the gym. |
|  |  | I should exercise and stretch the muscles in my arm on the side of my breast surgery regularly. |
|  |  | I should avoid using the arm on the side of my breast surgery to do any house chores. |
|  |  | I should avoid gaining weight because it can increase the risk of lymphedema. |
|  |  | I should wait a day to call my doctor if my arm suddenly becomes red and warm. |
|  |  | I should not use the arm on the side of my breast surgery to do light, repetitive office work. |
|  |  | I should not use the arm on the side of my breast surgery to do strenuous activity at work. |
